# Evidence-based therapist guided introduction to online heavy cannabis use treatment in Canadian adults: a Randomized Controlled Trial (RCT)

**DOI:** 10.1186/s42238-025-00378-5

**Published:** 2026-01-17

**Authors:** Karli K. Rysen, Julian M. Carusone, Jeffrey D. Wardell, Michael P. Schaub, Andreas Wenger, Harold Wallbridge, Jason D. Edgerton, Richard Kruk, Corey S. Mackenzie, Matthew T. Keough

**Affiliations:** 1 Present Address: Department of Psychology, University of Manitoba, 190 Dysart Rd, Winnipeg, MB R3T 2N2 Canada; 2 Department of Clinical Health Psychology, Max Rady College of Medicine, University of Manitoba, Winnipeg, Manitoba, Canada; 3 Department of Psychology, York University, Toronto, ON Canada; 4 Swiss Research Institute for Public Health and Addiction, University of Zurich, Zurich, Switzerland; 5 Department of Sociology and Criminology, University of Manitoba, Winnipeg, MB Canada; 6 Institute for Mental Health Policy Research, Centre for Addiction and Mental Health, Toronto, ON Canada; 7 Department of Psychiatry, University of Toronto, Toronto, ON Canada; 8 Senior Scientist, Homewood Research Institute, 150 Delhi St, Guelph, Ontario Canada

**Keywords:** Cannabis use, Cognitive behavioural therapy, Motivational enhancement therapy, Motivational interviewing, Online, Minimally guided

## Abstract

**Background:**

Many people who engage in heavy cannabis use do not seek treatment, and those who do are often met with long treatment wait times or high cost of services. Online treatment programs reduce barriers to accessing treatment in a timely manner. Online cannabis use treatment programs are effective, showing moderate effect sizes, particularly with text-based therapist support. Literature suggests brief therapist-guided introductions (i.e., self-completed interventions offered with the support of a therapist) informed by Motivational Enhancement Therapy (MET) may help to bolster and maintain program gains. The current evaluation of MET-informed therapist-guided introduction was conducted with a sample of Canadians who report heavy cannabis use, using a new Canadian version of CANreduce, an online treatment program for heavy cannabis use.

**Method:**

The intervention was pre-registered on clinicaltrials.gov for traceability (ID: NCT04965012). Participants (*N* = 152) were randomized into one of three conditions: MET-therapist guided introduction plus 6-week, online, self-guided treatment program; non-MET research assistant introduction plus 6-week, online, self-guided treatment program; or a psychoeducational control condition. Module content to reduce cannabis use was informed by cognitive behavioural therapy and motivational interviewing approaches. Participants completed assessments at baseline, end of treatment (i.e., 6 weeks), and at follow up (i.e., 10 weeks). Data were analyzed using Generalized Estimating Equations.

**Results:**

All participants reduced their cannabis consumption frequency (use days in the past week), as well as cannabis-related problems, at end of treatment and follow up. Participants in the MET-therapist condition showed significantly greater reductions in quantity of cannabis used over time compared to the waitlist control. Participants in the non-MET research assistant condition showed significantly greater reductions in cannabis problems compared to waitlist control. There were no significant differences between MET-therapist guided conditions and non-MET research assistant conditions. There was no significant effect of condition on cannabis consumption days in the past week, anxiety, depression or quality of life.

**Conclusion:**

The present study provides preliminary support for the CANreduce program in addition to the MET-therapist guided introduction.

**Supplementary Information:**

The online version contains supplementary material available at 10.1186/s42238-025-00378-5.

## Introduction

Before legalization of cannabis in October of 2018, cannabis was listed as the most commonly used illicit substance in Canada (Government of Canada 2017), with over half of all Canadians reporting having used cannabis at least once in their lifetime (Health Canada 2018). In the time since legalization, cannabis has continued to be a highly used psychoactive substance among Canadians, with approximately 5.2 million or 17% of Canadians over age 15 reporting using cannabis in the previous three months (Statistics Canada 2019). While many individuals can use cannabis recreationally and without major risks and consequences (Degenhardt & Hall 2001), some people use cannabis in a manner that puts them at higher risk of cannabis related risks and harms (Windle et al. 2019) such as negative physical effects of cannabis (Baggio et al. 2014; Tetrault et al. 2007), worsened psychiatric comorbidities (e.g., depression, anxiety, etc.; Arendt & Munk- Jørgensen, 2004; Fergusson et al. 2003; Khan et al. 2013; McLaren et al. 2010; Moore et al. 2007; Stefanis et al. 2004), interpersonal or social problems (APA, 2013; Copeland et al. 2001; Leos-Toro et al. 2017), and financial difficulties (Copeland et al. 2001). Cannabis misuse, broadly defined as the use of cannabis in frequent, chronic, or high quantity patterns, can substantially increase risk for associated harms compared to people who use cannabis casually (Calabria et al. 2010).

## Treatment approaches

There are a variety of treatment programs aimed at reducing cannabis consumption and mitigating the above-mentioned risks and harms associated with cannabis misuse. Literature consistently suggests that cognitive behavioural therapy (CBT) and motivational enhancement therapy (MET) are among the most efficacious online and in-person treatments for cannabis use, particularly when they are combined (Hoch et al. 2014; Gates et al. 2016; Rooke et al. 2013; Sabioni & Le Foll 2018). Effect sizes of combined CBT and MET programs range from small-to-large, with most falling in the moderate range (Hoch et al. 2014; Gates et al. 2016). McHugh et al. (2010) found that treatment for cannabis use disorder had larger treatment effect sizes compared to other substance use treatments (e.g., cocaine, opioids).

CBT is a treatment approach that aims to target maladaptive behavioural patterns leading to cannabis misuse, while addressing both motivational and cognitive barriers to change (McHugh et al. 2010). CBT is one of the most widely used forms of psychosocial treatment for substance use broadly (McHugh et al. 2010), and has been shown to be particularly efficacious in the treatment of cannabis misuse (Copeland et al. 2001; Sabioni & Le Foll 2018). However, CBT frameworks are rarely used as the sole intervention. While CBT can aid the individual to develop skills to reduce cannabis use and manage problems that may interfere with efforts to reduce cannabis use (Budney et al. 2007), it can be difficult for the individual with cannabis misuse to be engaged in treatment (e.g., lack of motivation, ambivalent about change).

CBT is most commonly combined with other frameworks, such as motivation interviewing/motivational enhancement therapy (MI/MET) to help increase engagement in treatment (Guydish et al. 2010).The goal of both MI and MET is to decrease ambivalence and increase motivation for individual change. Beginning a treatment program with MET has been shown to be efficacious in both reducing participant attrition (Philips & Wennberg 2014) and amplifying cannabis treatment outcomes (Gates et al. 2016). Carroll et al. (2001) demonstrated that participants in a substance use treatment program who received enhanced initial evaluation (i.e., MET) were significantly more likely to attend at least one additional treatment session. Indeed, Carroll et al. (2001) reported that 59% of participants receiving the enhanced initial evaluation continued the program, whereas only 29% of participants who did not receive the enhanced initial evaluation continued the program.

There is growing interest in shifting evidence-based treatments online for reasons such as cost-effectiveness (Budney et al. 2015), mitigating stigma (Richards & Viganó, 2013), and better access to treatment in a timely manner (Sibley & Weiner 2011), all of which serve to increase participation among individuals with cannabis use. Online modalities also help to bridge the gap between need and service when physical barriers to service exist, such as in remote and rural areas (Richards & Viganó, 2013), or during times where physical services are unavailable, such as the COVID-19 pandemic (Bergman & Kelly 2021). Existing literature on the efficacy of online interventions for wider substance misuse have been promising thus far (Elison et al. 2014; Sanchez & Bartel 2015; Trudeau et al. 2017), and evidence is growing to support online cannabis use treatment programs (Baumgartner et al. 2021; Budney et al. 2015; Jonas at el., 2018; Rooke et al. 2013; Schaub et al. 2015; Tait et al. 2013; Tossman et al., 2011). Several studies to date have also focused on evaluating the efficacy of online CBT and MET/MI treatment programs, given the demonstrated these modalities in an in-person format. Rooke and colleagues (2013) found that individuals who participated in an online, 6-module MI and CBT intervention reported fewer days of cannabis use, lower quantity of cannabis use and fewer symptoms of cannabis abuse compared to controls (i.e., individuals with access to web-based educational information) at a 6-week follow up, and reported fewer and less severe cannabis dependence than control at a 3-month follow up. However, the study by Rooke and colleagues (2013) reported issues with treatment adherence and retention of participants, where the average module completion was 3.5/6 modules, and their retention rate at follow up was 53%. Given that number of treatment modules completed was correlated with reduced frequency of use (Rooke et al. 2013), it may be important to find ways to promote and extend treatment adherence as much as possible in order to maximize and maintain treatment gains.

Of online treatment programs available, some are unguided (i.e., the participant completes the program independently; e.g., Rooke et al. 2013) and others are guided or semi-guided (i.e., the participant completes the program with varying levels of support from research personnel or therapists; e.g., Tossman et al., 2011). Current meta-analyses suggest that guided online treatment programs are generally more effective that unguided (Lam et al. 2024). Guided programs have mostly been supplemented with text-based support, where some find text-based counselling has no impact on cannabis-related treatment outcomes (e.g., Jonas et al., 2018), and others show positive impacts of text-based counselling (e.g., Schaub et al. 2015). CANreduce, an online, self-guided treatment program initially developed by Schaub and colleagues (2013) aims to help individuals reduce their cannabis use. The CANreduce program is an eight-module program that uses tenets of CBT, MI and behavioural self-management to help individuals learn to identify risky situations for use, manage cravings, deal with and prevent relapses, and learn strategies to maintain changes. In a 2015 study, Schaub and colleagues examined the efficacy of the CANreduce program with or without access to chat counselling during a six-week intervention period. Results from the study demonstrated that individuals in the CANreduce program with access to chat counselling reduced the frequency of their cannabis use significantly more than the CANreduce program without chat counselling or waitlist control (small effect size, *d* = 0.34), and also reduced the quantity of their cannabis use significantly more than the waitlist group (small effect size, *d* = 0.20).

In a subsequent study, CANreduce 2.0 was created to help overcome the issues of low intervention adherence and effectiveness. For CANreduce 2.0, Baumgartner and colleagues (2021) examined treatment outcomes for individuals across three treatment arms: completing the program with social presence (i.e., the semi-automated online coach, “Deborah”), completing the program with a service team (i.e., only anonymous semi-automated support, no “Deborah”) and waitlist control. Primary outcome results demonstrated reduced cannabis use days at three-month follow up across all groups (Baumgartner et al. 2021). Participants in both active treatment conditions (i.e., the social presence and service team conditions) reported significantly greater cannabis use reduction compared to control group immediately after treatment and at three-month follow up, with participants in the service team condition still reducing their cannabis-use days significantly more. There was no significant difference between the active conditions for cannabis use reductions. Baumgartner and colleagues (2021) also demonstrated differences in secondary outcomes: all groups decreased their Attention Deficit Hyperactivity Disorder (ADHD) self-report scores and depression without significant intergroup differences: however, both active treatment conditions (i.e., both service team and social presence team) showed greater reductions in general anxiety symptoms compared to control, and only the service team condition showed reduction in cannabis-use disorder severity, and severity of cannabis dependence compared to control.

Given that a participant’s perceived control to complete a treatment program predicts the number of treatment modules completed (Wojtowicz et al. 2013), and increased number of treatment sessions attended predicts better treatment outcomes (Copeland et al. 2001), bolstering motivation and autonomy at the outset of online programs may provide additional benefit beyond supportive-accountability. Specifically, integrating an individualized MET-therapist guided introduction to demonstrated effective online CBT programs, such as CANreduce, may increase participants’ feelings of self-efficacy and self-belief in change, in turn leading to better cannabis use outcomes. The MET-therapist introduction aims to act as a “primer” for subsequent CBT and MI heavy cannabis use treatment. Additionally, given that other online cannabis treatment programs have demonstrated that goal committment is the main predictor of treatment response (Jonas et al. 2019), exploring and setting individualized goals as part of an MET-introduction to treatment is important. To our knowledge, no such therapist guided introduction to a self-guided online cannabis treatment program exists.

## Aims and objectives

We took the *CANreduce* program originally tested in Switzerland by Schaub and colleagues (Amann et al. 2018; Baumgartner et al. 2021; Schaub et al., 2013, 2015) and adapted it for use in English-speaking Canadians. The present pilot randomized controlled trial (RCT) aims to examine the efficacy of an MET-therapist guided introduction to a Canadian version of the pre-established 2.0 CANreduce treatment program. We created two alternative guided introductions to the program: a therapist-guided introduction to the program using tenets of MET and CBT, as well as a research assistant-guided introduction to the program that contained no therapeutic components where the purpose was to solely show participants how to use the website. We conducted an RCT with two active treatment groups (i.e., MET therapist guided, non-MET research assistant guided) and one psychoeducational control group and obtained outcome data at both end of treatment (i.e., 6 weeks, T1) and follow up (i.e., 10 weeks, T2). The hypotheses and data analytic approach were modelled after the original Schaub et al., (2013, (Schaub, et al., 2015)) studies.

The hypotheses were as follows:

1. Hypothesis 1: Participants who receive either treatment program condition will have significant improvement in primary outcomes (i.e., reduced cannabis consumption days in the past week and quantity of cannabis) at the end of treatment (i.e., 6 weeks) and at follow up (i.e., 10 weeks) compared to the control condition, with participants in the MET therapist-guided introduction showing the greatest improvement in primary outcomes of interest.
2. Hypothesis 2: Participants who receive either treatment program condition will have significantly improved secondary outcomes of interest (i.e., lower cannabis-related problems, lower anxiety, lower depression, higher quality of life) at the end of treatment (i.e., 6 weeks) and at follow up (i.e., 10 weeks) compared to the control condition, with participants in the MET therapist-guided introduction showing the greatest improvement in secondary outcomes of interest.

## Method

### Design

The research was designed in accordance with the ethical principles of the Declaration of Helsinki and reported in accordance with the CONSORT guidelines for internet-based interventions (Eysenbach and Consort-EHEALTH Group, 2011), and was granted procedural ethics approval from the York University Office of Research Ethics (ORE) (Certificate # 2021–294) as well as the University of Manitoba Human Research Ethics Board (Protocol number HE2021-0145). The intervention was pre-registered on clinicaltrials.gov for traceability (ID: NCT04965012) and was updated at each stage of the research process.

The study was an open-label three-arm RCT. Participants were randomly assigned via random number generator to either the MET therapist-guided introduction (*n* = 63), the non-MET research assistant guided introduction (*n* = 52), or to the psychoeducational control condition (*n* = 37).^1^ Assessment data were collected at three distinct timepoints: baseline (i.e., T0), end of treatment (i.e., 6 weeks, T1) and follow-up (i.e., 10 weeks, T2). Participants received a $5 Amazon gift card for each assessment period they completed with an additional $5 Amazon gift card if they completed all three time-points, making the total compensation up to $20 Canadian Dollars. Researchers and participants were not blinded to group assignment. Participants who were eligible to receive student research participant credits at the University of Manitoba or York University also received participant pool credits for each time-point of participation.

## Procedure

### Participants

Participants were recruited from October 2022 to August 2023 using various strategies including online (e.g., Google Ads, Reddit), university-based (e.g., posters at university, student research participant pools), and community-based (e.g., posters at health clinics) methods.

Eligibility for the program included: 1) being over the age of 19, 2) currently residing in Ontario or Manitoba, 3) self-reporting difficulties with cannabis as indicated by a score of 8 or more on the Cannabis Use Disorders Identification Test – Revised (CUDIT-R; Adamson et al. 2010), 4) fluency in English, 5) having weekly Internet access with a device that allows for video connection, and 6) self-reporting at least a 6 out of 10 on a rating scale for motivation to reduce cannabis use. Participants were excluded if they self-reported 1) engaging in other psychological or pharmacological treatments for cannabis use, 2) elevated suicidality, as defined by scoring greater than minimal risk on the p4 suicidality screener (Dube et al. 2010), or 3) current serious psychiatric disorders or history of psychosis, schizophrenia, bipolar disorder. Informed consent for participation was provided electronically on the study website prior to registering for an account.

### Program overview

#### Treatment conditions

##### Active treatment conditions

Participants assigned to either of the active treatment conditions (i.e., the MET therapist-guided introduction or the non-MET research assistant-guided introduction) were contacted by email to arrange a time to meet with their assigned CANreduce facilitator via online videoconferencing platform. Participants in both of the active treatment conditions received access to the same version of the CANreduce treatment program, which was adapted from the newest version of the CANreduce treatment program (Amann et al. 2018), which is based on the original CANreduce treatment program by Schaub and colleagues (2013, 2015). The program was translated from German to English, modified to fit a Canadian context and altered to reflect a lower reading level (i.e., Flesch-Kincaid reading level of 7) to make it broadly applicable. The program is comprised of eight modules containing strategies of CBT and MI to help participants think about reasons for changing their cannabis use habits; consider benefits and harms of their current level of use, identify goals for cannabis use reduction; learn coping strategies for cravings, triggers, and social pressures; and learn how to prevent slips. Treatment modules also focus on building skills for the participants to better take care of themselves (i.e., better sleep schedules, reducing worry and rumination, and finding positive ways to cope with stress, like relying on friends and family). Participants were given six weeks to complete the eight modules and were encouraged to use the cannabis use diary (to help monitor and track their cannabis use) at a frequency of at least once per week.

##### MET-therapist guided introduction

The current study employed 10 graduate-level clinical psychology students from the University of Manitoba and York University as the program MET-therapists. Each MET-therapist received group training on MET, as well as additional training on the CANreduce program and MET-script prior to facilitating sessions. Ongoing MET-supervision was provided on a monthly basis, as well as individual supervision on an as-needed basis. Supervision was provided by two registered clinical psychologists and a senior clinical psychology PhD student trained in MET therapy. Adherence to MET treatment was assessed through live viewing of participant sessions by a supervisor, where a Treatment Fidelity Checklist (Borrelli 2011; Borrelli et al. 2015) was used to ensure at least 80% treatment fidelity (Borrelli et al. 2005). All five adherence checks passed, with an average score of 99% fidelity.

During the meeting, the participant and MET therapist completed the first module of the program together. The initial meeting took approximately one hour. The therapist’s discussion of the module was facilitated by an MET- and CBT-informed script which encouraged facilitating discussion about ambivalence to change, sourcing motivation, helping formulate initial cannabis reduction goals and providing normative feedback on the participant’s current level of cannabis use. Discussion also included a general overview of the program features, including how to use the cannabis use diary, where to locate information regarding mental health and crisis supports, and how to contact researchers for further inquiries or troubleshooting.

##### Non-MET research assistant guided introduction

The current study employed the skills of four undergraduate-level psychology students from York University. Each non-MET research assistant received individual training on the CANreduce program and the non-MET script prior to facilitating sessions. Non-MET research assistants also attended the monthly MET-supervision sessions, and particular care was given to addressing how to avoid integrating MET responses or questions into their sessions. Adherence to non-MET scripts for introduction were assessed through live viewing of participant sessions by a supervisor, where a Treatment Fidelity Checklist (Borrelli 2011; Borrelli et al. 2015) was used to ensure at least 80% treatment fidelity (Borrelli et al. 2005). All three adherence checks passed, with an average score of 91% fidelity.

During the meeting, the participant and non-MET research assistant briefly discussed the program without completing the first module of the program together. The initial meeting took approximately fifteen minutes. Discussion included a general overview of the program features, including how to use the cannabis use diary, where to locate information regarding mental health and crisis supports, and how to contact researchers for further inquiries or troubleshooting. The non-MET research assistants followed a script that contained no facets of MET and received ongoing training on how to respond to questions related to cannabis related use or goals in a non-therapeutic fashion.

##### Control condition

Participants assigned to the control group were given psychoeducational material for cannabis use (https://www.camh.ca/-/media/files/pdfs---reports-and-books---research/canadas-lower-risk-guidelines-cannabis-pdf.pdf) and well-being (e.g., https://cpa.ca/psychologyfactsheets/) that are readily available to the public, as is common practice for similar RCTs. At the 10-week mark (i.e., T2), participants were automatically given access to treatment modules and cannabis use diary, regardless of participation in T2 assessment, and offered a meeting with a non-MET research assistant if they were interested.

#### Engagement and accountability protocol

Engagement and accountability measures were implemented throughout the study process to maximize participant retention in the program and reduce participant drop out. Recommendations from standardized protocols for treatment retention (Gul & Ali 2010; Scott 2004; Wojtowicz et al. 2013) were adapted to the current study needs, which included extensively training staff (e.g., ensuring they are knowledgeable about the study population, teaching rapport building, minimizing bias and assumptions), explaining study procedures to participants in full (e.g., the reason for follow up studies, how collected information will be used) and various office procedures (e.g., tracking each contact with participants, flexible staffing to accommodate participant schedules, case review meetings). Other retention protocols also include using phone calls instead of emails where possible (Wojtowicz et al. 2013), having strong administrative teams with data coordinators (Gul & Ali 2010), and properly educating program workers responsible for first contact with participants are educated about the aim and process of the study (Gul & Ali 2010). All participants received reminders about upcoming introductory meetings the day prior. As a feature of the CANreduce program, participants received weekly automated emails for CANreduce tasks (e.g., weekly email to review progress and encourage participation; prompts to fill out diaries, reminders to fill out questionnaires at end of treatment), to encourage participation (e.g., lagging behind expected program completion, diaries not filled out) and offer personalized feedback based on cannabis use tracking (e.g., offering support or encouragement if cannabis use increased or decreased). Additionally, through the introductory meeting with either an MET therapist or non-MET research assistant, participants were able to ask questions about the program content, process or purpose at the outset, and were individually encouraged to email with any additional questions or problems. Finally, efforts to mitigate participant drop out included personal email and call reminders from research staff. Given that recruiting from a university sample and offering participant research credits can be associated with low long-term follow up, especially when spanning semesters where credits may no longer be required, Amazon gift cards were also offered to all participants for each time point of data collection.

### Measures

Participants completed all measures in the present study at all three time points (i.e., T0, T1 and T2) with the exception of the demographic questionnaire (T0 only) and Cannabis Use Disorders Identification Test (CUDIT-R; Adamson et al. 2010; T0 and T2 only). Data using other questionnaires assessing personality, attitudes towards treatment, suicidality, and other substance use were also collected as part of a larger study.

#### Primary outcomes

The primary outcomes were cannabis consumption days in the past week and quantity of cannabis use, as measured by the The Daily Sessions, Frequency, Age of Onset, and Quantity of Cannabis Use Inventory (DFAQ-CU; Cuttler & Spradlin 2017). Number of cannabis use days was assessed using a single question asking participants to indicate how many days in the past week they used cannabis ranging from 0 to 7 for all timepoints. The quantity of cannabis use was calculated (Range for T0 = 0—67.04; Range for T1 = 0—38.35; Range for T2 = 0—35.45) by multiplying the participant’s typical cannabis use per day by seven to calculate a weekly cannabis total estimate. Participants estimated their typical cannabis use per day referencing a standardized picture from the DFAQ-CU illustrating approximate weight in grams compared to an American dollar bill (Cuttler & Spradlin 2017). This method of standardization for cannabis use estimation has been used in similar studies (i.e., Schaub et al. 2015).

#### Secondary outcomes

##### Cannabis problems

Cannabis problems were assessed at all time points using the Rutger’s Marijuana Problem Index (RMPI; White et al. 2005). Sum scores were calculated (Range for T0 = 1–51; Range for T1 = 0—39; Range for T2 = 0—39), and the RMPI internal consistency over three time points ranged from good to excellent (α = 0.86—0.90).

##### Depression

Depression was assessed at all time points using the Patient Health Questionnaire (PHQ-9; Kroenke et al. 2001). Sum scores were calculated (Range for T0 = 0—27; Range for T1 = 0—25; Range for T2 = 0—27) and the PHQ-9 internal consistency over three time points ranged from good to excellent (α = 0.88–0.92).

##### Anxiety

Anxiety was assessed at all time points using the Generalized Anxiety Disorder Scale (GAD-7; Spitzer et al. 2006). Sum scores were calculated (Range for T0 = 0—21; Range for T1 = 0—21; Range for T2 = 0—21), and the GAD-7 internal consistency over three time points was excellent (α = 0.90–0.94).

##### Quality of life

Quality of life was measured with the World Health Organization Quality of Life Assessment (WHOQOL-BREF, WHOQOL Group 1998). The questionnaire includes 26 self-report items that assess quality of life in four distinct domains (Range for T0 = 42—114; Range for T1 = 61—107; Range for T2 = 50—115). The reliabilities of each subscale were: physical health ranged from questionable to good (α = 0.69—0.81), psychological ranged from acceptable to good (α = 0.74–0.81), social relationships ranged from acceptable to good (α = 0.76–0.85), and environment ranged from acceptable to good (α = 0.78–0.80).

#### Other measures

##### Demographics

Demographic information was collected from participants at T0 to determine eligibility and describe the sample. Information included age, sex, gender, ethnicity, employment status, history and treatment for physical or mental health conditions, and family history of cannabis misuse.

##### Motivation for change

Information regarding motivation for change was collected at T0 (for screening purposes), as well as during the beginning and end of both the individual MET-therapist session and the non-MET research assistant condition. Motivation for change included a three-item questionnaire assessing motivation for change across the domains of importance (Range for T0 = 6—10; Range for T1 = 1—10; Range for T2 = 1—10), confidence (Range for T0 = 1—10; Range for T1 = 1—10; Range for T2 = 1—10) and readiness (Range for T0 = 2—10; Range for T1 = 1—10; Range for T2 = 1—10). Participants rated their motivation from 1 (not important/confident/ready) to 10 (very important/confident/ready). A score of 6 or greater was required on the importance subscale to be included in the study.

##### Cannabis use disorder severity

The Cannabis Use Disorders Identification Test-Revised (CUDIT-R; Adamson et al. 2010) was used to assess cannabis use disorder severity at baseline. The CUDIT-R is an 8-item self-report measure. Sum scores were calculated, where higher scores indicated more problematic cannabis use. A cut off of 8 on this measure was used for inclusion criteria for the study. The internal consistency of the CUDIT-R at baseline was questionable (α = 0.68).

### Statistical analysis

#### Power

Effect sizes reported for cannabis misuse treatment range from small-to-large, varying among different modalities and delivery methods of treatment. For combined CBT & MET/MI treatments, some studies report moderate-to-large effect sizes (Gates et al. 2016; Hoch et al. 2014). In comparison, studies examining online intervention of cannabis use found small but significant effect sizes (Rooke et al. 2013; Tait et al. 2013). The Swiss CANreduce treatment program by Schaub et al. (2015) found small effect sizes (*d* = 0.20 for self-help with chat versus waitlist, and *d* = 0.34 for self-help with chat versus self-help without chat). Given the wide array of effect sizes across similar studies, it is expected that the current intervention will yield small-to-medium effect sizes. A Power Analysis was conducted for a 3 (within; time) by 3 (between; group) mixed design. Using G*Power, the sample required to detect a small effect with 80% power, *α* = 0.05, and a correlation of 0.60 between repeated measures was 135.

#### Data analytic plan

Data was analyzed using SPSS version 25.0. First, preliminary analyses were run to assess normality, baseline differences, missing data and descriptives to characterize the study sample. These preliminary analyses allowed for observing any potential systematic missingness, which allowed for the inclusion of relevant covariates in the main analyses (Enders, 2010).

We used Generalized Estimating Equations (GEE) within an intent-to-treat (ITT; Gupta 2011) model, where we included all participants who were randomized and responded to at least one email from the research team regardless of level of participation in the program. Missing data was treated with full maxiumum likelihood estimation. For each of the main analyses, we used separate mixed models to examine the effects of time (Coded as 0, 1 and 2; within-subjects), intervention (between-subjects) and intervention by time interaction on the primary and secondary outcomes. The trend for time was linear, random intercepts (but not random slopes) were specified, and all outcomes were treated as continuous. Distributions of outcomes were normal. We examined distributions for all variables across time for primary and secondary outcomes, and all of these variables had acceptable levels of skewness and kurtosis (skew < 3, kurtosis < 10; Kline 2011). A relevant covariate (i.e., baseline anxiety) based on the missing data analysis (see below in results section) and important pre-treatment factors (i.e., age, history of mental illness diagnosis, and baseline cannabis use severity) were included in the models with the goal of reducing potential biases (Preacher et al. 2010).

## Results

### Descriptive statistics and missing data analysis

A total of 808 participants were initially screened for participation, but 656 did not meet the eligibility criteria or did not respond to any contact from the CANreduce team and were not included. This resulted in a final sample of 152 participants (*M*_age_ = 30.20, *SD*_age_ = 10.50, 58.6% female) in the trial, which was composed of treatment completers and non-treatment completers, as we used an intent to treat (ITT) model. Of this sample, individuals identified as 65.8% White, 7.9% South Asian, 5.9% Indigenous, 5.9% Middle Eastern, North African, or Central Asian, 5.3% Black, 3.3% East/Southeast Asian or Pacific Islander, 1.3% Hispanic or Latino, and 3.3% specified other.

**Fig. 1:**
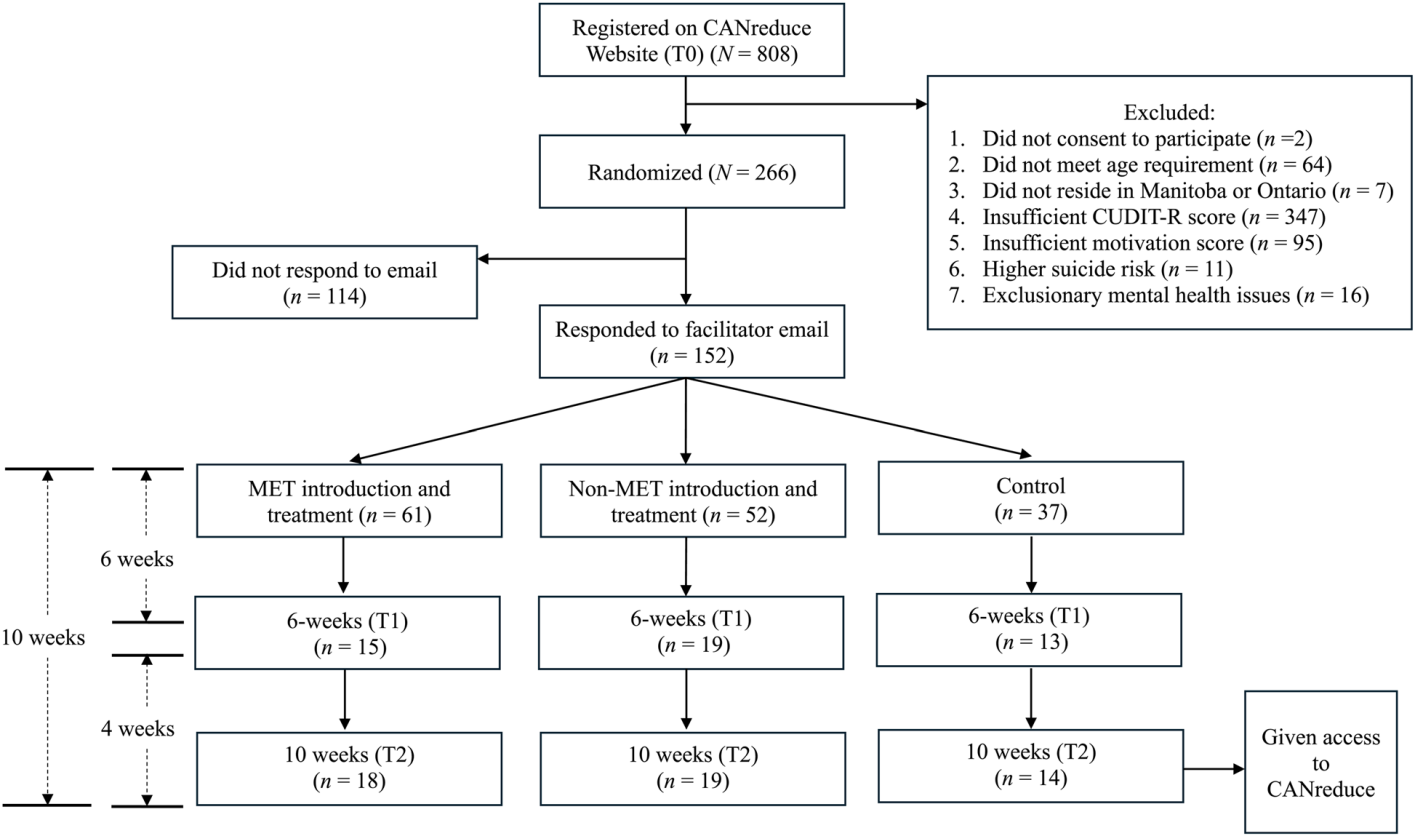
CONSORT Trial Flow Chart

See Fig. 1 for the CONSORT trial flow chart. Demographic information for each of the three conditions is presented in Supplementary Table 1 and can be found in Supplementary material. A large portion (*n* = 347) of the recruited individuals did not meet the cut off for hazardous cannabis use (as indicated with a score of less than 8 on the CUDIT-R). This is unsurprising given the recruitment method of university participant pool, where students could gain credit for completing questionnaires regardless of cannabis use or interest in the CANreduce program. Other reasons for ineligibility for the program included not consenting to participate in the research (*n* = 2), being under 19 years of age (*n* = 64), not residing in a province where CANreduce was currently being offered (*n* = 7), insufficient motivation score (as indicated with a score less than 6 on the importance for change scale; *n* = 95), more than minimal suicide risk (*n* = 11) or having a current/historical exclusionary mental health issue (*n* = 16).

Despite study procedures to mitigate drop out (e.g., accountability protocols at sign up, automatic reminders, compensation, etc.), attrition rates at T2 were greater than expected. We predicted that attrition rates at 10 weeks would be approximately 35% attrition rate based on literature and the addition of retention protocols. Our overall dropout rate was 66% between T0 and T2 (i.e., number of participants completing baseline primary outcome measures dropped from 150 at baseline to 51 at follow up; 34% retention rate). Broken down by condition, 28% of participants in the MET-therapist condition remained at T2 (72% attrition), 37% of participants in the non-MET research assistant condition remained at T2 (63% attrition), and 38% of participants in the waitlist control condition remained at T2 (62% attrition). We did not observe any significant differences on attrition by condition (*F*(2, 149) = 0.600, *p* = 0.550).

The overall dropout rate is identical to a similarly structured study examining the efficacy of an online self-guided program, *Take Care of Me,* by Frohlich and colleagues (2022) which addresses comorbid alcohol use and emotional problems, which also saw a 66% attrition rate between T0 and T2. Significant attrition is purportedly observed widely among e-health studies (Eysenbach 2005) and internet-based substance use treatment (e.g., 35% retention rate; Etter 2005).

For individuals that completed the introductory meeting with either the MET-therapist or non-MET research assistant, the average percent of the program completed was 51.58% (*SD* = 36.82) with 24.5% (*n* = 23) completing all 8 modules. Comparing the MET-therapist and non-MET research assistant groups (for only individuals who attended the introductory session, as only these individuals had access to the program) using t-test revealed that individuals did not significantly complete more modules across conditions (*t*(92) = −0.899, *p* = 0.185, Cohen’s *d* = −0.19).

**Fig. 2:**
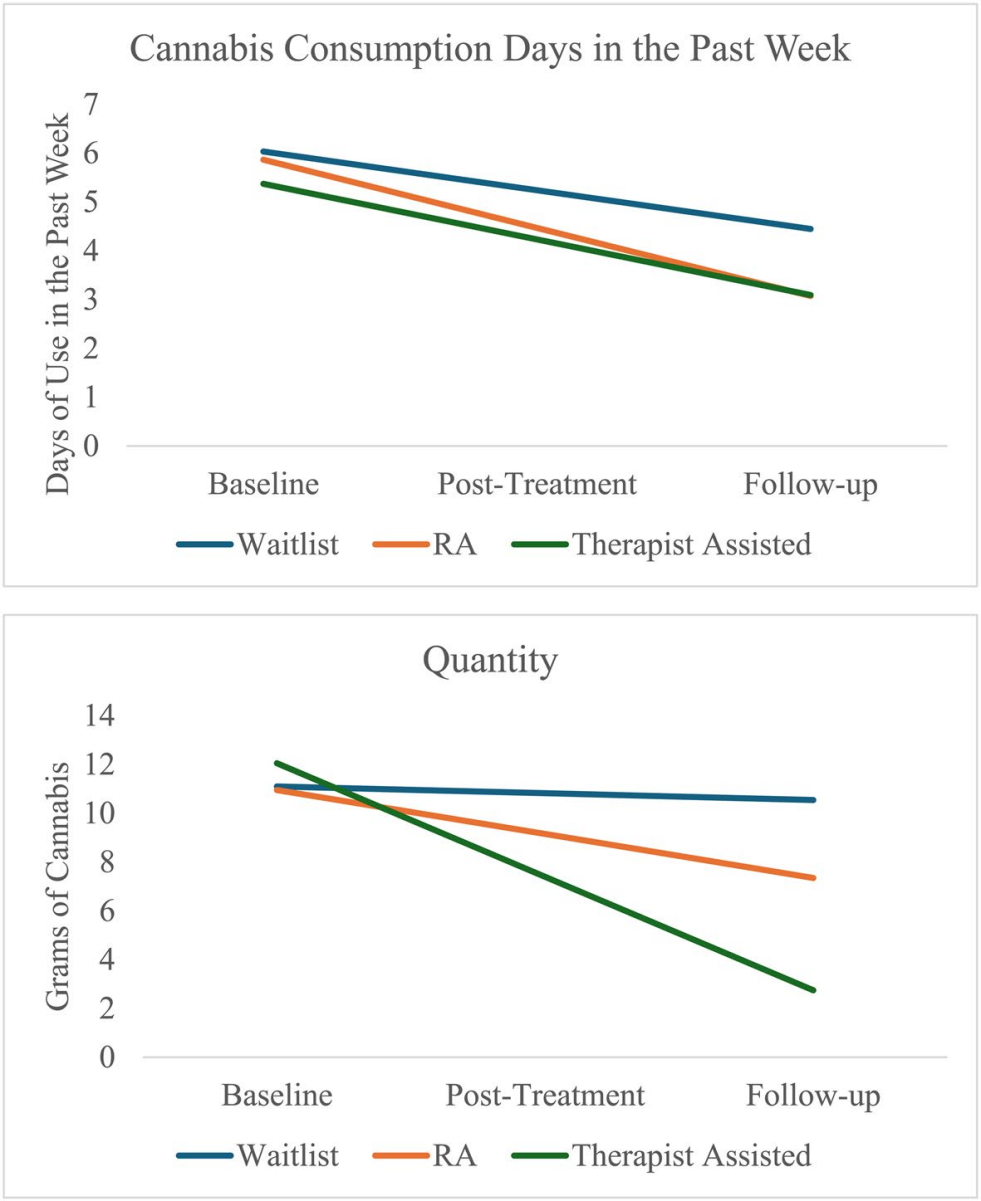
Changes in primary outcomes (Cannabis consumption days in the past week and quantity) over time. Note: Quantity Time x Condition interactions were significant for waitlist versus therapist condition (*p* = 0.01)

Regressions were used to examine relevant auxiliary variables that accounted for missingness. The dichotomous missingness variable was included in Step 1, and relevant covariates (i.e., age, history of mental illness diagnosis, and baseline cannabis use severity; Lev-Ran et al. 2013; Rajapaksha et al. 2020; Statistics Canada 2018) were included in Step 2. Missing data emerged as a significant predictor of baseline scores in Step 1 for cannabis consumption days in the past week (*R*^2^ = 0.183, *F*(4, 140) = 7.821, *p* < 0.001), cannabis problems (*R*^2^ = 0.436, *F*(4, 128) = 24.768, *p* < 0.001), anxiety (*R*^2^ = 0.190, *F*(4, 142) = 8.351, *p* < 0.001), depression (*R*^2^ = 0.163, *F*(4, 142) = 6.933, *p* < 0.001), and quality of life (*R*^2^ = 0.134, *F*(4, 133) = 5.159, *p* < 0.001) but not cannabis quantity (*R*^2^ = 0.050, *F*(4, 131) = 0.1.709, *p* = 0.152). After inclusion of the relevant covariates (i.e., age, history of mental illness diagnosis, and baseline cannabis use severity) most effects became non-statistically significant in Step 2 (i..e, cannabis consumption days in the past week (*p* = 0.768); problems (*p* = 0.431); depression (*p* = 0.110); quality of life (*p* = 0.116)). However, the missing effect on anxiety remained statistically significant (*p* = 0.024). Therefore, baseline anxiety, age, history of mental illness diagnosis and baseline cannabis use disorder severity were also included as relevant covariates in the GEE models (fig. 2).

### Main trial analyses

#### Hypothesis 1: Treatment effects on the primary outcomes

##### Cannabis consumption days in the past week

There was a significant main effect of time, where cannabis consumption days in the past week decreased over time for participants in all conditions (*B* = −0.79, *SE* = 0.32, *p* = 0.01). *Overall*, we did not observe expected follow-up treatment effects on cannabis consumption days in the past week. The time by condition interaction was not significant for the waitlist control versus non-MET research assistant condition (*B* = −0.60, *SE* = 0.49 *p* = 0.22), the waitlist control versus the MET-therapist condition (*B* = −0.35, *SE* = 0.44, *p* = 0.44), and between the non-MET research condition versus the MET-therapist condition (*B* = 0.26, *SE* = 0.49, *p* = 0.60). Results indicated that all three of the groups significantly decreased their cannabis consumption days in the past week, though none of the groups significantly differed from each other. See Table 1 for details.

**Table 1:** Post-Hoc GEE Model results for primary cannabis consumption days in the past week outcome

Parameter	*B*	Std. Error	df	Wald x^2^	Sig
*Primary* C*annabis Consumption Days in the Past Week Outcome*					
Intercept	6.03	0.46	1	174.33	.000
Time	−0.79	0.32	1	6.28	.012
Anxiety covariate	0.02	0.03	1	0.22	.640
CUD severity score covariate	0.16	0.04	1	17.49	.000
Mental illness diagnosis covariate	0.70	0.34	1	4.16	.041
Age covariate	0.11	0.05	1	5.03	.025
Waitlist vs non-MET	−0.17	0.37	1	0.20	.649
Waitlist vs MET	−0.66	0.40	1	2.74	.098
Time xWaitlist vs non-MET	−0.60	0.49	1	1.53	.216
Time x Waitlist vs MET	−0.35	0.44	1	0.61	.436

*Note.* Primary cannabis outcome variables for the cannabis consumption days in the past week (as measured by the DFAQ) over the course of the study (i.e., T0-T2). Waitlist denotes waitlist control, non-MET denotes the non-MET research assistant condition, and MET denotes the MET-therapist condition

##### Cannabis quantity

We found partial support for expected follow up treatment effects on cannabis quantity. The time by condition interaction was not significant for waitlist control versus non-MET research assistant condition (*B* = −1.52, *SE* = 2.10, *p* = 0.47), and the non-MET research condition versus the MET-therapist condition (*B* = −2.85, *SE* = 1.88, *p* = 0.13), but the interaction was significant for waitlist control versus the MET-therapist condition (*B* = −4.37, *SE* = 1.75, *p* = 0.01). Results indicated that participants who received MET-therapist treatment (*B* = 12.05, *SE* = 2.78, *p* < 0.001) showed significantly greater reductions in the quantity of cannabis used relative to the waitlist control. See Table 2 for details (Fig. 2).

**Table 2:** Post-Hoc GEE Model results for primary quantity outcome

Parameter	*B*	Std. Error	df	Wald x^2^	Sig
*Primary Quantity Outcome*					
Intercept	11.10	3.34	1	11.02	.001
Time	−0.28	1.51	1	0.03	.854
Anxiety covariate	−0.13	0.16	1	0.64	.424
CUD severity score covariate	0.32	0.18	1	3.28	.070
Mental illness diagnosis covariate	1.80	1.72	1	1.10	.295
Age covariate	−0.75	0.36	1	4.33	.037
Waitlist vs non-MET	−0.15	2.84	1	0.00	.958
Waitlist vs MET	0.95	2.66	1	0.13	.721
Time x Waitlist vs non-MET	−1.52	2.10	1	0.53	.469
**Time x Waitlist vs MET**	**−4.37**	**1.75**	**1**	**6.23**	**.013**

*Note.* Primary cannabis outcome variables for the quantity (DFAQ) over the course of the study (i.e., T0-T2). Waitlist denotes waitlist control, non-MET denotes the non-MET research assistant condition, and MET denotes the MET-therapist condition

#### Hypothesis 2: Immediate effects on secondary outcomes

##### Cannabis problems

**Fig. 3:**
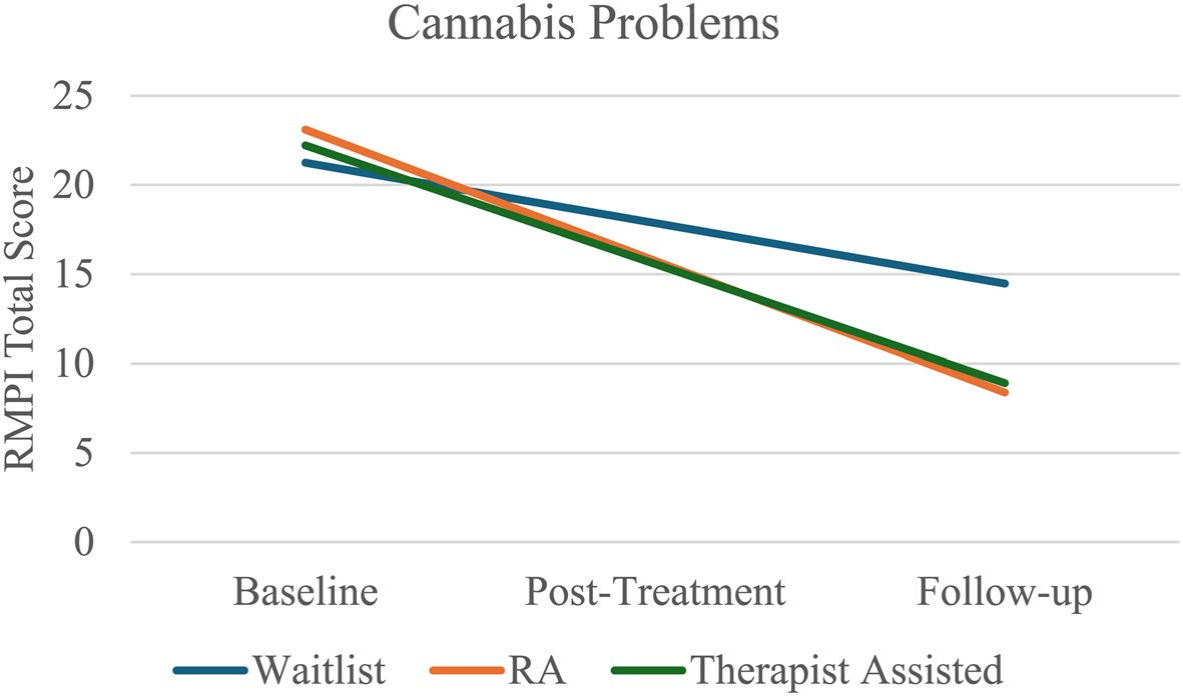
Changes in significant secondary outcomes (Cannabis Problems, Over Time). Note: Problems Time x Condition interactions were significant for RA versus waitlist (*p* =.03) and marginally significant for therapist versus waitlist (*p* =.06)

There was a significant main effect of time, where cannabis problems decreased over time (*B* = −3.39, *SE* = 1.12, *p* = 0.002). We found partial support for expected follow up treatment effects on cannabis-related problems. The time by condition interaction was not significant for the non-MET research condition versus the MET-therapist condition (*B* = 0.71, *SE* = 1.93, *p* = 0.72), and for the time by condition interaction for the waitlist control versus the MET-therapist condition (*B* = −3.26, *SE* = 1.75, *p* = 0.06). Although the latter was not significant, it approached significance. Hence, for descriptive reasons we probed the interaction, but use caution when interpreting it given that the interaction was not statistically significant. The time by condition interaction was significant for the waitlist control versus non-MET research assistant condition (*B* = −3.97, *SE* = 1.80, *p* = 0.03). Results indicated that although all conditions decreased their cannabis-related problems over time, participants who received the non-MET research assistant condition (*B* = 23.10, *SE* = 1.83, *p* < 0.001) significantly reduced the amount of cannabis use problems compared to waitlist control. Participants who received the MET-therapist treatment (*B* = 12.05, *SE* = 2.78, *p* < 0.001) also decreased their cannabis use problems compared to waitlist control, though not to a traditionally significant amount (i.e., *p* value less than 0.05). See Table 3 for details (fig. 3).

**Table 3:** Post-Hoc GEE Model results for cannabis problems

Parameter	*B*	Std. Error	df	Wald x^2^	Sig
*Secondary Problems Outcome*					
Intercept	21.25	2.07	1	104.92	.000
Time	−3.39	1.12	1	9.17	.002
Anxiety covariate	0.34	0.13	1	7.15	.007
CUD severity score covariate	0.88	0.14	1	40.51	.000
Mental illness diagnosis covariate	1.32	1.54	1	0.74	.390
Age covariate	0.02	0.17	1	0.01	.924
Waitlist vs non-MET	1.86	1.82	1	1.04	.308
Waitlist vs MET	0.97	1.71	1	0.32	.569
**Time x Waitlist vs non-MET**	**−3.97**	**1.80**	**1**	**4.88**	**.027**
**Time x Waitlist vs MET**	**−3.26**	**1.75**	**1**	**3.49**	**.062**

*Note.* Secondary cannabis outcome variables for the cannabis problems (RMPI) over the course of the study (i.e., T0-T2). Waitlist denotes waitlist control, non-MET denotes the non-MET research assistant condition, and MET denotes the MET-therapist condition

### Anxiety, depression and quality of life

We did not observe any main effect of time, or interaction effects, on the remaining secondary outcomes of anxiety, depression, or quality of life. See Tables 4, 5 and 6 for more details.

**Table 4:** Post-Hoc GEE Model results for anxiety

Parameter	*B*	Std. Error	df	Wald x^2^	Sig
*Anxiety*					
Intercept	11.78	1.00	1	139.30	<.001
Time	−0.83	0.68	1	1.48	.224
CUD severity score covariate	0.36	0.09	1	17.74	<.001
Mental illness diagnosis covariate	2.28	0.94	1	5.82	.016
Age covariate	−0.03	0.13	1	0.04	.846
Waitlist vs non-MET	−0.59	1.27	1	0.22	.642
Waitlist vs MET	−0.27	1.23	1	0.05	.829
Time x Waitlist vs non-MET	−1.43	0.89	1	2.58	.108
Time x Waitlist vs MET	−0.90	1.08	1	0.70	.403

*Note.* Secondary outcome variables for anxiety (GAD7) over the course of the study (i.e., T0-T2). Anxiety not included as covariate. Waitlist denotes waitlist control, non-MET denotes the non-MET research assistant condition, and MET denotes the MET-therapist condition

**Table 5:** Post-Hoc GEE Model results for depression

Parameter	*B*	Std. Error	df	Wald x^2^	Sig
*Depression*					
Intercept	7.25	1.13	1	41.31	.000
Time	−1.79	0.95	1	3.52	.061
Anxiety covariate	0.58	0.07	1	62.10	.000
CUD severity score covariate	0.10	0.08	1	1.46	.227
Mental illness diagnosis covariate	1.50	0.82	1	3.29	.070
Age covariate	0.05	0.11	1	0.21	.644
Waitlist vs non-MET	−0.45	0.93	1	0.23	.631
Waitlist vs MET	−1.36	1.00	1	1.86	.173
Time x Waitlist vs non-MET	−0.47	1.20	1	0.16	.694
Time x Waitlist vs MET	0.47	1.25	1	0.14	.707

*Note.* Secondary cannabis outcome variables for depression (PHQ-9) over the course of the study (i.e., T0-T2). Waitlist denotes waitlist control, non-MET denotes the non-MET research assistant condition, and MET denotes the MET-therapist condition

**Table 6:** Post-Hoc GEE Model results for quality of life

Parameter	*B*	Std. Error	df	Wald x^2^	Sig
*Quality of Life*					
Intercept	85.44	2.82	1	915.51	.000
Time	2.38	2.03	1	1.38	.241
Anxiety covariate	−0.51	0.18	1	8.00	.005
CUD severity score covariate	−0.33	0.22	1	2.33	.127
Mental illness diagnosis covariate	−2.03	2.28	1	0.80	.372
Age covariate	−0.35	0.34	1	1.06	.304
Waitlist vs non-MET	1.13	2.79	1	0.16	.687
Waitlist vs MET	4.68	2.84	1	2.72	.099
Time x Waitlist vs non-MET	1.60	2.98	1	0.29	.592
Time x Waitlist vs MET	−0.14	2.69	1	0.00	.959

*Note.* Secondary cannabis outcome variables for quality of life (WHOQOL) over the course of the study (i.e., T0-T2). Waitlist denotes waitlist control, non-MET denotes the non-MET research assistant condition, and MET denotes the MET-therapist condition

## Discussion

While online programs for cannabis use are relatively new in Canada, available evidence suggests that combined CBT and MI programs have moderate effect sizes in reducing cannabis related outcomes. The present pilot RCT aimed to examine the efficacy of a Canadian version of an established Swiss self-help program, CANreduce, as well as any additional benefit of an MET-therapist guided introduction to the program. Although there was significant attrition and challenges with overall sample size, the present study provides preliminary support for both the Canadian version of CANreduce and the benefit of the MET-therapist guided introduction.

Regarding primary outcomes, we saw partial support for our first hypothesis. While there was a general reduction in cannabis consumption days in the past week from baseline to follow up across conditions, there were no significant differences between groups (i.e., either active treatment group compared to waitlist control, or the other active treatment group). A general reduction in cannabis consumption not surprising, given that the present sample was that of a treatment seeking population and was likely to begin modifying their cannabis use with or without the CANreduce program. However, in examining quantity of cannabis use, participants in the MET-therapist condition showed significantly greater reductions in quantity of cannabis used over time compared to the waitlist control. These findings are in part similar to previous iterations of the CANreduce program, where the present study demonstrated significantly greater reductions in cannabis use quantity for the conditions where therapeutic interactions were present compared to waitlist control (i.e., the MET-therapist condition in the present study and the group that had access to chat counselling in Schaub et al. 2015). However, the present study differed from the Schaub et al. (2015) study in that the present study did not also see significant reduction of cannabis use days in the past week. It is possible that the expected reduction of cannabis use days in the past week was not found either due to our small sample size reducing the ability to detect meaningful changes, or by using a broad measure of frequency (i.e., number of days used in the past week) instead of a more sensitive measure (e.g., number of smoking sessions in the past week). It is also possible that because the CANreduce program allowed for flexible goal making (either general cannabis reduction *or* abstinence), individuals who successfully reduced their overall quantity of use did not have the goal of having cannabis-free days, hence did not show a reduction in number of cannabis use days in the past week.

Regarding secondary outcomes, we again saw only partial support for our second hypothesis. Significant differences for secondary outcomes between study conditions were only found for cannabis-related problems. Although participants across groups all decreased their cannabis-related problems from baseline to follow up, only participants in the non-MET research assistant condition showed significantly greater reductions in cannabis-related problems compared to the waitlist control. Participants in the MET-therapist condition also showed a reduction in cannabis use problems over time compared to the waitlist control, albeit just above the traditional cut off for significance (i.e., *p* = 0.06). However, given the relatively small sample size, we may have been underpowered to elucidate these differences within this sample, giving reasonable support to further investigate significance values close to the traditional cutoff. This finding is similar to prior CANreduce studies, where the present study demonstrated significant reductions in cannabis use problems for the non-therapeutic support compared to waitlist control (i.e., the non-MET research assistant condition in the present study, and service team support in the Baumgartner and colleagues (2021) study). While the present study and the Baumgartner and colleagues (2021) examined slightly different constructs of cannabis associated problems (i.e., Baumgartner outcomes included cannabis use disorder and cannabis-dependence severity; the present study examined cannabis-related problems directly), they do examine a similar general construct of cannabis-related problems. This finding of significance in the case of the non-MET research assistant condition and the nearly-significant trend in the case of the MET-therapist condition in relation to the waitlist control group, indicate that access to the online treatment program itself is helpful in reducing cannabis-related problems. Given that a diagnosis of Cannabis Use Disorder (CUD) reflects an overall level of impairment to functioning and cannabis-related problems rather than cannabis consumption days in the past week or quantity of cannabis use itself, the demonstrated improvement in cannabis-related problems supports the efficacy of the Canadian version of CANreduce for those with CUD.

Somewhat surprisingly, no condition differences were found for anxiety, depression or quality of life. Prior iterations of the Swiss CANreduce found support for reduction in anxiety (Baumgartner et al. 2021), and similarly structured research in other substance use areas have typically found support for reduction in negative emotions (i.e., depression and anxiety) and quality of life (Frohlich et al. 2022). The absence of findings for reduction in anxiety, depression and quality of life is also somewhat surprising given the relationship reported in the literature between improvement in cannabis misuse and well-being (Tossman et al. 2011; Hser et al. 2017). However, as suggested in other short-term cannabis reduction studies examining quality of life as an outcome, a relatively short survey period may not be adequate time to fully capture changes to quality of life which may take longer to improve (Hser et al. 2017). Prior CANreduce studies had a follow up time of three months (Schaub et al. 2015; Amann et al. 2018) instead of the Canadian CANreduce follow up of only one month, which may have limited our ability to observe quantifiable positive impacts of cannabis use changes. Additionally, the current Canadian CANreduce had limited focus specifically on mental health-related constructs, whereas other similar substance use treatment program evaluations more heavily integrated mental health and well-being information in their treatment modules and tracked this domain on a week-to-week basis (Frohlich et al. 2022). Similar to primary outcomes, we were likely underpowered to observe some of the changes in secondary outcomes.

### Implications

Overall, our study offers preliminary support for the Canadian CANreduce, an online self-guided treatment program for cannabis use. While our results are modest and sample size is relatively small, the current findings offer some additional support to the growing literature supporting online cannabis use treatments (Baumgartner et al,. 2021; Rooke et al. 2013; Schaub et al. 2015). Given the increasing cost of in-person treatment, limited resources paired with increased demand and reduced access in remote and rural areas (Richards & Viganó, 2013), increasing the availability of evidence-based online treatments for cannabis use is much needed. Available literature suggests that online-delivered CBT/MET treatment for CUD is equally effective to therapist-delivered CBT/MET but online-delivered are significantly more cost-effective (Budney et al. 2015). Given that potential substance use treatment patients can be lost if treatment is not available or readily accessible (National Institute on Drug Abuse 2018), timely access to treatment is important. CANreduce offers an alternative approach to addressing heavy cannabis use that may help reduce the time between being ready for treatment and receiving treatment.

Additionally, the present study was the first of its kind to develop and integrate a one-hour guided introduction to the program alongside an MET-trained therapist. Prior literature has detailed the benefits of including MET principles at the outset of substance use treatment (Carroll et al. 2001), while also highlighting the significant strain of individualized treatment on the healthcare system (Morgan et al. 2013). Offering a hybrid approach may help to promote balance between the cost-effective online treatments while “priming” the participant to get the most out of the programming available. Despite the modest beneficial outcomes of the MET-therapist condition compared to the non-MET research assistant condition in the domain of quantity of cannabis use, we cannot in confidence champion the use of MET-guided introductions over non-MET introductions. More research is needed to better explore the areas of benefit of MET-guided introductions, the long-term impacts, as well as further exploring the cost/benefit analysis of more intensive resources required for the MET-therapist conditions.

### Limitations and future directions

The present study is not without limitations. First, the present study experienced significant attrition and disengagement despite attempts to maximize participation (e.g., adherence protocols, automatic reminders, offering ongoing technical support). Relatedly, participants tended to complete only approximately half of module content offered. Despite weekly automated progress and diary reminders, the proportion of participants who fully completed the program was 24.5%, with no significant completion differences between the two treatment groups. This is similar to other online cannabis treatments (e.g., 3.5/6 modules, Rooke et al. 2013). Although significant participant drop out and disengagement is not uncommon in any online substance use treatment (Hadjistavropoulos et al. 2020; Frohlich et al. 2022; Rooke et al. 2013), future research should continue to refine practices to maximize retention. Strategies such as implementing tenets of contingency management (e.g., giving additional positive reinforcement for completing modules, reinforcing progress towards cannabis reduction or abstinence goals), fully implementing the Engagement, Verification, Maintenance and Confirmation (EVMC) Protocol by Scott (2004), or having scheduled virtual progress check ins with participants instead of offering email progress summaries, may help to maximize retention. Given the integration of the CANreduce program with MET-therapists, participants may gain additional benefit from multiple check ins throughout treatment with the MET-therapist. While there is some evidence to suggest that number of treatment sessions does not impact treatment effect size (Davis et al. 2015), multiple check ins with a consistent MET-therapist may foster the therapeutic alliance, which can significantly impact cannabis use and problematic behaviours at post-treatment follow up (Diamond et al. 2006). Furthermore, while we attempted to correct for biases associated with baseline levels of anxiety, we did observe that people who were more anxious at the outset of the treatment trial were more likely to drop out over time. Future trials on CANreduce should enhance the anxiety-specific content.

Second, the current study’s smaller sample size limited our options in post-hoc analyses. Conditions under which the study was run (i.e., in the context of a Ph.D. dissertation project with limited budget and restricted time for data collection) prevented larger sample sizes from being recruited. We could not examine whether treatment completers had significantly different primary and secondary outcomes of interest compared to non-completers. Along the same line, the current study’s small sample size also limited the ability to examine how various sociodemographic and individual factors related to treatment outcomes. Given that individuals respond differentially to treatment based on various personal factors (Dacosta-Sanchez et al. 2023), future studies should aim to increase sample size through larger data collection projects in addition to mitigating drop out. The small sample size also significantly impacted study power as mentioned throughout the manuscript, as well as generalizability of study results.

## Conclusions

CANreduce is one of the first online, CBT and MET self-guided treatment programs for heavy cannabis use available in North America that has an integrated MET-therapist guided introduction. We found preliminary evidence suggesting the guided introduction’s version of the program efficacy in reducing cannabis quantity, alongside the general benefit of reducing cannabis-related problems among both the non-guided introduction and the guided introduction (albeit, with marginal significance). In spite of the significant study attrition and small sample size, the current study offers support to the growing literature for online treatments for heavy cannabis use. Future studies should aim to replicate these findings with a larger sample, examine the efficacy among more varied levels of cannabis users (i.e., not only individuals with hazardous or disordered use), and find the optimal level of therapist engagement for a mostly-self guided program.

## Supplementary Information

Supplementary Material 1.

## Data Availability

The participants of this study did not give written consent for their data to be shared publicly, so due to the sensitive nature of the research supporting data is not available.

## Abbreviations

ADHD: Attention Deficit Hyperactivity Disorder
CBT: Cognitive behavioural therapy
CUD: Cannabis Use Disorder
GEE: Generalized Estimating Equations
ITT: Intent-to-treat
MET: Motivational Enhancement therapy
MI: Motivational Interviewing
RCT: Randomized Controlled Trial

## Footnotes

1 Note participants were originally assigned at a 1:1:1 randomization across conditions. The listed *n* values represent numbers from an Intent To Treat framework after randomization, hence the unequal values. See Fig. 1 for more information.

## References

[CR1] Adamson SJ, Kay-Lambkin FJ, Baker AL, Lewin TJ, Thornton L, Kelly BJ, et al. An improved brief measure of cannabis misuse: the Cannabis Use Disorders Identification Test-Revised (CUDIT-R). Drug Alcohol Depend. 2010;110(1–2):137–43. 20347232 10.1016/j.drugalcdep.2010.02.017

[CR2] American Psychiatric Association. Diagnostic and statistical manual of mental disorders. 5th ed. 2013. 10.1176/appi.books.9780890425596.

[CR3] Amann M, Haug S, Wenger A, Baumgartner C, Ebert DD, Berger T, et al. The effects of social presence on adherence-focused guidance in problematic cannabis users: protocol for the CANreduce 2.0 randomized controlled trial. JMIR Res Protoc. 2018;7(1):e9484. 10.2196/resprot.9484 PMC5812982 29386176

[CR4] Arendt M, Munk-Jørgensen P. Heavy cannabis users seeking treatment. Soc Psychiatry Psychiatr Epidemiol. 2004;39(2):97–105. 15052390 10.1007/s00127-004-0719-7

[CR5] Baggio S, N’Goran AA, Deline S, Studer J, Dupuis M, Henchoz Y, et al. Patterns of cannabis use and prospective associations with health issues among young males. Addiction. 2014;109(6):937–45. 24450535 10.1111/add.12490

[CR6] Baumgartner C, Schaub MP, Wenger A, Malischnig D, Augsburger M, Walter M, et al. CANreduce 2.0 adherence-focused guidance for internet self-help among cannabis users: three-arm randomized controlled trial. J Med Internet Res. 2021;23(4):e27463. 33929333 10.2196/27463 PMC8122293

[CR7] Bergman BG, Kelly JF. Online digital recovery support services: an overview of the science and their potential to help individuals with substance use disorder during COVID-19 and beyond. J Subst Abuse Treat. 2021;120:108152. 33129636 10.1016/j.jsat.2020.108152 PMC7532989

[CR8] Borrelli B. The assessment, monitoring, and enhancement of treatment fidelity in public health clinical trials. J Public Health Dent. 2011;71:S52–63. 21656954

[CR9] Borrelli B, Sepinwall D, Ernst D, Bellg AJ, Czajkowski S, Breger R, et al. A new tool to assess treatment fidelity and evaluation of treatment fidelity across 10 years of health behavior research. J Consult Clin Psychol. 2005;73(5):852. 16287385 10.1037/0022-006X.73.5.852

[CR10] Borrelli B, Tooley EM, Scott-Sheldon LA. Motivational interviewing for parent-child health interventions: a systematic review and meta-analysis. Pediatr Dent. 2015;37(3):254–65. 26063554

[CR11] Budney AJ, Roffman R, Stephens RS, Walker D. Marijuana dependence and its treatment. Addict Sci Clin Pract. 2007;4(1):4. 18292704 10.1151/ascp07414 PMC2797098

[CR12] Budney AJ, Stanger C, Tilford JM, Scherer EB, Brown PC, Li Z, et al. Computer-assisted behavioral therapy and contingency management for cannabis use disorder. Psychol Addict Behav. 2015;29(3):501. 25938629 10.1037/adb0000078 PMC4586287

[CR13] Calabria B, Degenhardt L, Hall W, Lynskey M. Does cannabis use increase the risk of death? Systematic review of epidemiological evidence on adverse effects of cannabis use. Drug Alcohol Rev. 2010;29(3):318–30. 20565525 10.1111/j.1465-3362.2009.00149.x

[CR14] Carroll KM, Libby B, Sheehan J, Hyland N. Motivational interviewing to enhance treatment initiation in substance abusers: an effectiveness study. Am J Addict. 2001;10(4):335–9. 11783748 10.1080/aja.10.4.335.339 PMC3680596

[CR15] Copeland J, Swift W, Roffman R, Stephens R. A randomized controlled trial of brief cognitive–behavioral interventions for cannabis use disorder. J Subst Abuse Treat. 2001;21(2):55–64. 11551733 10.1016/s0740-5472(01)00179-9

[CR16] Cuttler C, Spradlin A. Measuring cannabis consumption: psychometric properties of the daily sessions, frequency, age of onset, and quantity of cannabis use inventory (DFAQ-CU). PLoS ONE. 2017;12(5):e0178194. 28552942 10.1371/journal.pone.0178194 PMC5446174

[CR17] Dacosta-Sánchez D, Fernández-Calderón F, Blanc-Molina A, Díaz-Batanero C, Lozano OM. Monitoring adherence and abstinence of cannabis use disorder patients: profile identification and relationship with long-term treatment outcomes. J Subst Use Addict Treat. 2023;148:209019. 36933660 10.1016/j.josat.2023.209019

[CR18] Davis ML, Powers MB, Handelsman P, Medina JL, Zvolensky M, Smits JA. Behavioral therapies for treatment-seeking cannabis users: a meta-analysis of randomized controlled trials. Eval Health Prof. 2015;38(1):94–114. 24695072 10.1177/0163278714529970 PMC4429893

[CR19] Degenhardt L, Hall W. The association between psychosis and problematical drug use among Australian adults: findings from the National Survey of Mental Health and Well-Being. Psychol Med. 2001;31(4):659. 11352368 10.1017/s0033291701003865

[CR20] Diamond GS, Liddle HA, Wintersteen MB, Dennis ML, Godley SH, Tims F. Early therapeutic alliance as a predictor of treatment outcome for adolescent cannabis users in outpatient treatment. Am J Addict. 2006;15:s26–33. 10.1080/10550490601003664 17182417

[CR21] Dube P, Kroenke K, Bair MJ, Theobald D, Williams LS. The P4 screener: evaluation of a brief measure for assessing potential suicide risk in 2 randomized effectiveness trials of primary care and oncology patients. Prim Care Companion CNS Disord. 2010;12(6):27151. 10.4088/PCC.10m00978blu PMC3067996 21494337

[CR22] Elison S, Humphreys L, Ward J, Davies G. A pilot outcomes evaluation for computer assisted therapy for substance misuse–an evaluation of breaking free online. J Subst Use. 2014;19(4):313–8.

[CR23] Enders C. Applied missing data analysis. New York: Guilford Press; 2010.

[CR24] Etter JF. Comparing the efficacy of two Internet-based, computer-tailored smoking cessation programs: a randomized trial. J Med Internet Res. 2005;7(1):e373. 10.2196/jmir.7.1.e2 PMC1550632 15829474

[CR25] Eysenbach G. The law of attrition. J Med Internet Res. 2005;7(1):e402. 10.2196/jmir.7.1.e11 PMC1550631 15829473

[CR26] Eysenbach G, Consort-EHEALTH Group. CONSORT-EHEALTH: improving and standardizing evaluation reports of web-based and mobile health interventions. J Med Internet Res. 2011;13(4):e1923. 10.2196/jmir.1923 PMC3278112 22209829

[CR27] Fergusson DM, Horwood LJ, Swain-Campbell NR. Cannabis dependence and psychotic symptoms in young people. Psychol Med. 2003;33(1):15–21. 12537032 10.1017/s0033291702006402

[CR28] Frohlich JR, Rapinda KK, Schaub MP, Wenger A, Baumgartner C, Johnson EA, et al. Examining differential responses to the take care of me trial: a latent class and moderation analysis. Addict Behav Rep. 2022;16:100437. 35694108 10.1016/j.abrep.2022.100437 PMC9184289

[CR29] Gates, P. J., Sabioni, P., Copeland, J., Le Foll, B., & Gowing, L. Psychosocial interventions for cannabis use disorder. Cochrane Database of Systematic Reviews 2016;5. 10.1002/14651858.CD005336.pub4 PMC4914383 27149547

[CR30] Government of Canada, 2017. Summary results for 2017 Canadian Tobacco, Alcohol and Drugs Survey. From: https://www.canada.ca/en/health-canada/services/canadian-tobacco-alcohol-drugs-survey/2017-summary.html, Accessed date: 24 July 2020

[CR31] Gul RB, Ali PA. Clinical trials: the challenge of recruitment and retention of participants. J Clin Nurs. 2010;19(1–2):227–33. 20500260 10.1111/j.1365-2702.2009.03041.x

[CR32] Guydish J, Jessup M, Tajima B, Manser ST. Adoption of motivational interviewing and motivational enhancement therapy following clinical trials. J Psychoact Drugs. 2010;42(6):215–26. 10.1080/02791072.2010.10400545 PMC3349358 21138198

[CR33] Hadjistavropoulos HD, Mehta S, Wilhelms A, Keough MT, Sundström C. A systematic review of internet-delivered cognitive behavior therapy for alcohol misuse: study characteristics, program content and outcomes. Cogn Behav Ther. 2020;49(4):327–46. 31599198 10.1080/16506073.2019.1663258

[CR34] Health Canada (2017). Canadian Tobacco, Alcohol and Drugs (CTADS) Survey: 2017 detailed tables. Available at: https://www.canada.ca/en/health-canada/services/canadian-tobacco-alcohol-drugs-survey/2017-summary/2017-detailed-tables.html#t16.

[CR35] Hoch E, Bühringer G, Pixa A, Dittmer K, Henker J, Seifert A, et al. Candis treatment program for cannabis use disorders: findings from a randomized multi-site translational trial. Drug Alcohol Depend. 2014;134:185–93. 24176199 10.1016/j.drugalcdep.2013.09.028

[CR36] Hser YI, Mooney LJ, Huang D, Zhu Y, Tomko RL, McClure E, et al. Reductions in cannabis use are associated with improvements in anxiety, depression, and sleep quality, but not quality of life. J Subst Abuse Treat. 2017;81:53–8. 28847455 10.1016/j.jsat.2017.07.012 PMC5607644

[CR37] Gupta SK. Intention-to-treat concept: a review. Perspect Clin Res. 2011;2(3):109–12. 21897887 10.4103/2229-3485.83221 PMC3159210

[CR38] Jonas B, Tensil MD, Leuschner F, Strüber E, Tossmann P. Predictors of treatment response in a web-based intervention for cannabis users. Internet Interv. 2019;18:100261. 31890614 10.1016/j.invent.2019.100261 PMC6926274

[CR39] Khan SS, Secades-Villa R, Okuda M, Wang S, Pérez-Fuentes G, Kerridge BT, et al. Gender differences in cannabis use disorders: results from the National epidemiologic survey of alcohol and related conditions. Drug Alcohol Depend. 2013;130(1–3):101–8. 23182839 10.1016/j.drugalcdep.2012.10.015 PMC3586748

[CR40] Kline RB. Principles and practice of structural equation modeling (3rd ed.). Guilford Press 2011.

[CR41] Kroenke K, Spitzer RL, Williams JB. The PHQ-9: validity of a brief depression severity measure. J Gen Intern Med. 2001;16(9):606–13. 11556941 10.1046/j.1525-1497.2001.016009606.x PMC1495268

[CR42] Lam, R. W., Kennedy, S. H., Adams, C., Bahji, A., Beaulieu, S., Bhat, V., ... & Milev RV. Canadian Network for Mood and Anxiety Treatments (CANMAT) 2023 Update on Clinical Guidelines for Management of Major Depressive Disorder in Adults: Réseau canadien pour les traitements de l'humeur et de l'anxiété (CANMAT) 2023: Mise à jour des lignes directrices cliniques pour la prise en charge du trouble dépressif majeur chez les adultes. The Canadian Journal of Psychiatry, 2024;07067437241245384. 10.1177/07067437241245384 PMC11351064 38711351

[CR43] Lev-Ran S, Le Foll B, McKenzie K, George TP, Rehm J. Cannabis use and cannabis use disorders among individuals with mental illness. Compr Psychiatry. 2013;54(6):589–98. 23375264 10.1016/j.comppsych.2012.12.021

[CR44] Leos-Toro C, Rynard V, Hammond D. Prevalence of problematic cannabis use in Canada: cross-sectional findings from the 2013 Canadian tobacco, alcohol and drugs survey. Can J Public Health. 2017;108(5–6):e516–22. 10.17269/CJPH.108.5955 PMC6972268 29356658

[CR45] McHugh RK, Hearon BA, Otto MW. Cognitive behavioral therapy for substance use disorders. Psychiatr Clin North Am. 2010;33(3):511–25. 20599130 10.1016/j.psc.2010.04.012 PMC2897895

[CR46] McLaren JA, Silins E, Hutchinson D, Mattick RP, Hall W. Assessing evidence for a causal link between cannabis and psychosis: a review of cohort studies. Int J Drug Policy. 2010;21(1):10–9. 19783132 10.1016/j.drugpo.2009.09.001

[CR47] Moore TH, Zammit S, Lingford-Hughes A, Barnes TR, Jones PB, Burke M, et al. Cannabis use and risk of psychotic or affective mental health outcomes: a systematic review. Lancet. 2007;370(9584):319–28. 17662880 10.1016/S0140-6736(07)61162-3

[CR48] Morgan TB, Crane DR, Moore AM, Eggett DL. The cost of treating substance use disorders: individual versus family therapy. J Fam Ther. 2013;35(1):2–23.

[CR49] National Institute on Drug Abuse (2018). Principles of Drug Addiction Treatment: A Research-Based Guide (Third edition). Retrieved from: https://www.drugabuse.gov/download/675/principles-drug-addiction-treatment-research-based-guide-third-edition.pdf?v=87ecd1341039d24b0fd616c5589c2095

[CR50] Philips B, Wennberg P. The importance of therapy motivation for patients with substance use disorders. Psychother. 2014;51(4):555. 10.1037/a0033360 24059740

[CR51] Preacher KJ, Zyphur MJ, Zhang Z. A general multilevel SEM framework for assessing multilevel mediation. Psychol Methods. 2010;15(3):209. 20822249 10.1037/a0020141

[CR52] Rajapaksha RMDS, Hammonds R, Filbey F, Choudhary PK, Biswas S. A preliminary risk prediction model for cannabis use disorder. Prev Med Rep. 2020;20:101228. 33204605 10.1016/j.pmedr.2020.101228 PMC7649639

[CR53] Richards D, Viganó N. Online counseling: a narrative and critical review of the literature. J Clin Psychol. 2013;69(9):994–1011. 23630010 10.1002/jclp.21974

[CR54] Rooke S, Copeland J, Norberg M, Hine D, McCambridge J. Effectiveness of a self-guided web-based cannabis treatment program: randomized controlled trial. J Med Internet Res. 2013;15(2):e26. 23470329 10.2196/jmir.2256 PMC3636012

[CR55] Sabioni P, Le Foll B. Psychosocial and pharmacological interventions for the treatment of cannabis use disorder. F1000Res. 2018. 10.12688/f1000research.11191.1. 29497498 10.12688/f1000research.11191.1 PMC5811668

[CR56] Sanchez RP, Bartel CM. The feasibility and acceptability of “Arise”: an online substance abuse relapse prevention program. Games Health J. 2015;4(2):136–44. 26181807 10.1089/g4h.2014.0015 PMC4601551

[CR57] Scott CK. A replicable model for achieving over 90% follow-up rates in longitudinal studies of substance abusers. Drug Alcohol Depend. 2004;74(1):21–36. 15072804 10.1016/j.drugalcdep.2003.11.007 PMC5937263

[CR58] Schaub MP, Haug S, Wenger A, Berg O, Sullivan R, Beck T, Stark L. Can reduce-the effects of chat-counseling and web-based self-help, web-based self-help alone and a waiting list control program on cannabis use in problematic cannabis users: A randomized controlled trial. BMC Psychiatry, 2013;13(1):305. 10.1186/1471-244X-13-305 PMC3830542 24228630

[CR59] Schaub MP, Wenger A, Berg O, Beck T, Stark L, Buehler E, et al. A web-based self-help intervention with and without chat counseling to reduce cannabis use in problematic cannabis users: three-arm randomized controlled trial. J Med Internet Res. 2015;17(10):e232. 26462848 10.2196/jmir.4860 PMC4642392

[CR60] Sibley LM, Weiner JP. An evaluation of access to health care services along the rural-urban continuum in Canada. BMC Health Serv Res. 2011;11(1):20. 21281470 10.1186/1472-6963-11-20 PMC3045284

[CR61] Spitzer RL, Kroenke K, Williams JB, Löwe B. A brief measure for assessing generalized anxiety disorder: the GAD-7. Arch Intern Med. 2006;166(10):1092–7. 16717171 10.1001/archinte.166.10.1092

[CR62] Statistics Canada. (2018). National Cannabis Survey, first quarter 2018. Retrieved October 29, 2018 from: https://www150.statcan.gc.ca/n1/daily-quotidien/180418/dq180418b-eng.htm.

[CR63] Statistics Canada (2019). National Cannabis Survey, third quarter 2019. Retrieved July 24, 2020 from: https://www150.statcan.gc.ca/n1/daily-quotidien/191030/dq191030a-eng.htm.

[CR64] Stefanis NC, Delespaul P, Henquet C, Bakoula C, Stefanis CN, Van Os J. Early adolescent cannabis exposure and positive and negative dimensions of psychosis. Addiction. 2004;99(10):1333–41. 15369572 10.1111/j.1360-0443.2004.00806.x

[CR65] Tait RJ, Spijkerman R, Riper H. Internet and computer based interventions for cannabis use: a meta-analysis. Drug Alcohol Depend. 2013;133(2):295–304. 23747236 10.1016/j.drugalcdep.2013.05.012

[CR66] Tetrault JM, Crothers K, Moore BA, Mehra R, Concato J, Fiellin DA. Effects of marijuana smoking on pulmonary function and respiratory complications: a systematic review. Arch Intern Med. 2007;167(3):221–8. 17296876 10.1001/archinte.167.3.221 PMC2720277

[CR67] Tossmann DHP, Jonas B, Tensil MD, Lang P, Strüber E. A controlled trial of an internet-based intervention program for cannabis users. Cyberpsychol Behav Soc Netw. 2011;14(11):673–9. 21651419 10.1089/cyber.2010.0506

[CR68] Trudeau KJ, Black RA, Kamon JL, Sussman S. A randomized controlled trial of an online relapse prevention program for adolescents in substance abuse treatment. Child Youth Care Forum. 2017;46:437–54. 10.1007/s10566-016-9387-5.

[CR69] White HR, Labouvie EW, Papadaratsakis V. Changes in substance use during the transition to adulthood: a comparison of college students and their noncollege age peers. J Drug Issues. 2005;35(2):281–306.

[CR70] WHOQOL Group. Development of the World Health Organization WHOQOL-BREF quality of life assessment. Psychol Med. 1998;28(3):551–8. 10.1017/s0033291798006667. 9626712 10.1017/s0033291798006667

[CR71] Windle SB, Wade K, Filion KB, Kimmelman J, Thombs BD, Eisenberg MJ. Potential harms from legalization of recreational cannabis use in Canada. Can J Public Health. 2019;110(2):222–6. 30759307 10.17269/s41997-018-00173-1 PMC6964625

[CR72] Wojtowicz M, Day V, McGrath P. Predictors of participant retention in a guided online self-help program for university students: prospective cohort study. J Med Internet Res. 2013;15(5):e96. 10.2196/jmir.2323. 23697614 10.2196/jmir.2323 PMC3668607

