# Supplementary material for: Evidence-based therapist guided introduction to online heavy cannabis use treatment in Canadian adults: a Randomized Controlled Trial (RCT)
Source: J Cannabis Res. 2026 Jan 17;8:26. doi: 10.1186/s42238-025-00378-5 (PMC12896346; doi:10.1186/s42238-025-00378-5)
Supplement: Supplementary file 1 — Supplementary Material 1. [file 42238_2025_378_MOESM1_ESM.docx]

**Table 1**

*Descriptive Statistics of Study Variables by Group at Baseline*

| Variable | Intervention | | |
| --- | --- | --- | --- |
|  | MET-therapist  (*n* = 63) | Non-MET research assistant (*n =* 52) | Control (*n =* 37) |
| Age, *M* (SD) | 31.14 (11.42) | 30.63 (11.71) | 27.97 (7.32) |
| Sex, % (*n*) |  |  |  |
| Male | 33.3 (21) | 42.3 (22) | 50 (18) |
| Female | 65.1 (41) | 57.7 (30) | 50 (18) |
| Intersex | 1.6 (1) | 0 (0) | 0 (0) |
| Ethnicity, % (*n*) |  |  |  |
| East Asian, South-East Asian, Pacific Islander (e.g., Chinese, Japanese, Korean, Vietnamese, Thai) | 3.2 (2) | 5.8 (3) | 0 (0) |
| Middle Eastern, North African, Central Asian (e.g., Jordanian, Saudi, Egyptian, Moroccan, Iranian) | 4.8 (3) | 7.7 (4) | 5.7 (2) |
| Hispanic or Latino (e.g., Brazilian, Chilean, Mexican, Cuban) | 0 (0) | 1.9 (1) | 2.9 (1) |
| Caucasian or White (e.g., Russian, German, Latvian, French, Scottish, Italian) | 63.5 (40) | 69.2 (36) | 68.6 (24) |
| Black (e.g., African- American, Nigerian, Haitian, Jamaican, Somali) | 4.8 (3) | 5.8 (3) | 5.7 (2) |
| Indigenous or Aboriginal (e.g., First Nations, Inuit, Metis, Native American, Native Australian) | 11.1 (7) | 3.8 (2) | 0 (0) |
| South Asian (e.g., Indian, Pakistani, Sri Lankan, Nepalese) | 9.5 (6) | 3.8 (2) | 8.6 (3) |
| Other | 3.2 (2) | 0 (0) | 8.6 (3) |
| Cannabis cannabis consumption days in the past week, *M* (SD) | 5.66 (2.39) | 5.90 (2.03) | 6.24 (1.69) |
| Cannabis grams/week, *M* (SD) | 11.02 (14.71) | 9.95 (14.76) | 11.49 (14.07) |
| RMPI, *M* (SD) | 26.36 (10.82) | 26.82 (10.64) | 26.14 (10.34) |
| GAD-7, *M* (SD) | 11.95 (6.28) | 10.98 (6.21) | 11.78 (6.34) |
| PHQ-9, *M* (SD) | 14.27 (6.62) | 12.38 (6.52) | 13.73 (7.21) |
| QOL, *M* (SD) | 79.80 (13.16) | 83.92 (12.02) | 79.97 (16.36) |
| Mental Illness Diagnosis, % (*n*) |  |  |  |
| No | 33.3 (21) | 45.1 (23) | 48.6 (17) |
| Yes | 66.7 (42) | 54.9 (28) | 51.4 (18) |

*Note.* RMPI = Rutgers Marijuana Problems Index; GAD-7 = Generalized Anxiety Disorder scale; PHQ-9 = Patient Health Questionnaire scale; QOL = World Health Organization Quality of Life Assessment.
